# From functional annotation to functional meaning in microbiome research

**DOI:** 10.1186/s40793-026-00956-x

**Published:** 2026-09-02

**Authors:** Rajesh Kumar Bajiya, Rania Agabi, Jimena Olabarría García, Vicenzo Paolo Torreno, Jean Armengaud, Lucia Grenga

**Affiliations:** 1 Université Paris-Saclay, CEA, INRAE, Département Médicaments et Technologies pour la Santé (DMTS), SPI, Bagnols-sur-Cèze, 30200 France; 2 Université Montpellier, Montpellier, 34090 France; 3 University of Rennes, Rennes, 35000 France; 4 Infrastructure Nationale de Protéomique, ProFI, UAR 2048, Bagnols-sur-Cèze, France

**Keywords:** Microbiome function, Functional annotation, Functional inference, Functional capacity, Functional realization, Functional impact, Systems microbiology

## Abstract

Microbiome research has moved from cataloging community composition to asking what these communities do, but “function” is often used to describe fundamentally different levels of evidence. Functional claims may refer to functional capacity (what is encoded), functional realization (what molecular functions are actively engaged under specific conditions), or functional impact (the resulting consequences for hosts, microbial communities, or ecosystems). In this Perspective, we examine how functional annotations in microbiome research generate biological meaning and why current approaches support different kinds of inference. We propose a framework that distinguishes three levels of functional inference: capacity, realization, and impact. Homology-based, domain-centric, pathway-based, machine learning-driven, and multi-omics approaches each contribute differently to these levels, but none alone captures microbiome function in full. We argue that microbiome function should be interpreted as a hierarchy of inferences rather than a single property. Recognizing this distinction should improve how functional findings are interpreted, reported, and compared across microbiome studies. Accordingly, functional studies should explicitly state whether their conclusions concern capacity, realization, or impact, thereby clarifying the evidential basis of functional claims and improving their interpretation and comparison across microbiome studies.

**Supplementary Information:**

The online version contains supplementary material available at https://doi.org/10.1186/s40793-026-00956-x.

## Why “function” is hard to define in microbiome research

Microbiome research has progressively shifted from describing community composition to inferring biological function [1–3]. Yet in microbiome research, “function” is not a single property but a set of distinct inferences spanning functional capacity (what is encoded), functional realization (what molecular functions are actively engaged under specific conditions), and functional impact (the resulting consequences for hosts, microbial communities, or ecosystems). These are related but not equivalent claims, and much of the conceptual confusion in the field comes from treating them as interchangeable.

This transition has been mediated by functional annotation, in which genes, proteins, or pathways detected in sequencing data are assigned biological meaning by projection onto curated reference databases. Increasingly, these annotations are integrated into pathway-centric, genome-scale, and ecological representations that describe microbiomes as interconnected biochemical systems rather than collections of individual taxa [4, 5]. But because these descriptions are inferred rather than directly observed, they do not all describe the same level of biological evidence.

Functional annotation can be biased when the organisms under study are distantly related to model organisms, as simple annotation transfer based solely on sequence similarity tends to propagate and sometimes amplify errors across related branches of the Tree of Life [6, 7]. Besides, functional annotation remains constrained by incomplete and unevenly distributed reference knowledge, leaving substantial portions of microbiome-encoded functions unresolved [8–10]. Moreover, metabolic pathways are often distributed across community members, such that pathway reconstruction reflects inferred community-level capacity rather than coordinated organism-level activity. Similar functional outputs may also arise depending on environmental conditions, host context, and interspecies interactions [11–15]. At the molecular level, many biologically relevant microbial metabolites and extracellular products remain difficult to identify, quantify, or mechanistically interpret [16].

These limitations do not diminish the value of functional profiling approaches; rather, they emphasize the need for more explicit frameworks for interpreting what different types of functional data actually represent. In this perspective, we examine how function is currently inferred through annotation frameworks, what types of biological knowledge these approaches produce, and where their interpretative limits lie. We propose a conceptual framework distinguishing functional capacity, realization, and impact, and use it to evaluate the strengths and limitations of current annotation strategies and guide more biologically meaningful functional interpretation.

## How functional inference is generated

**Fig. 1:**
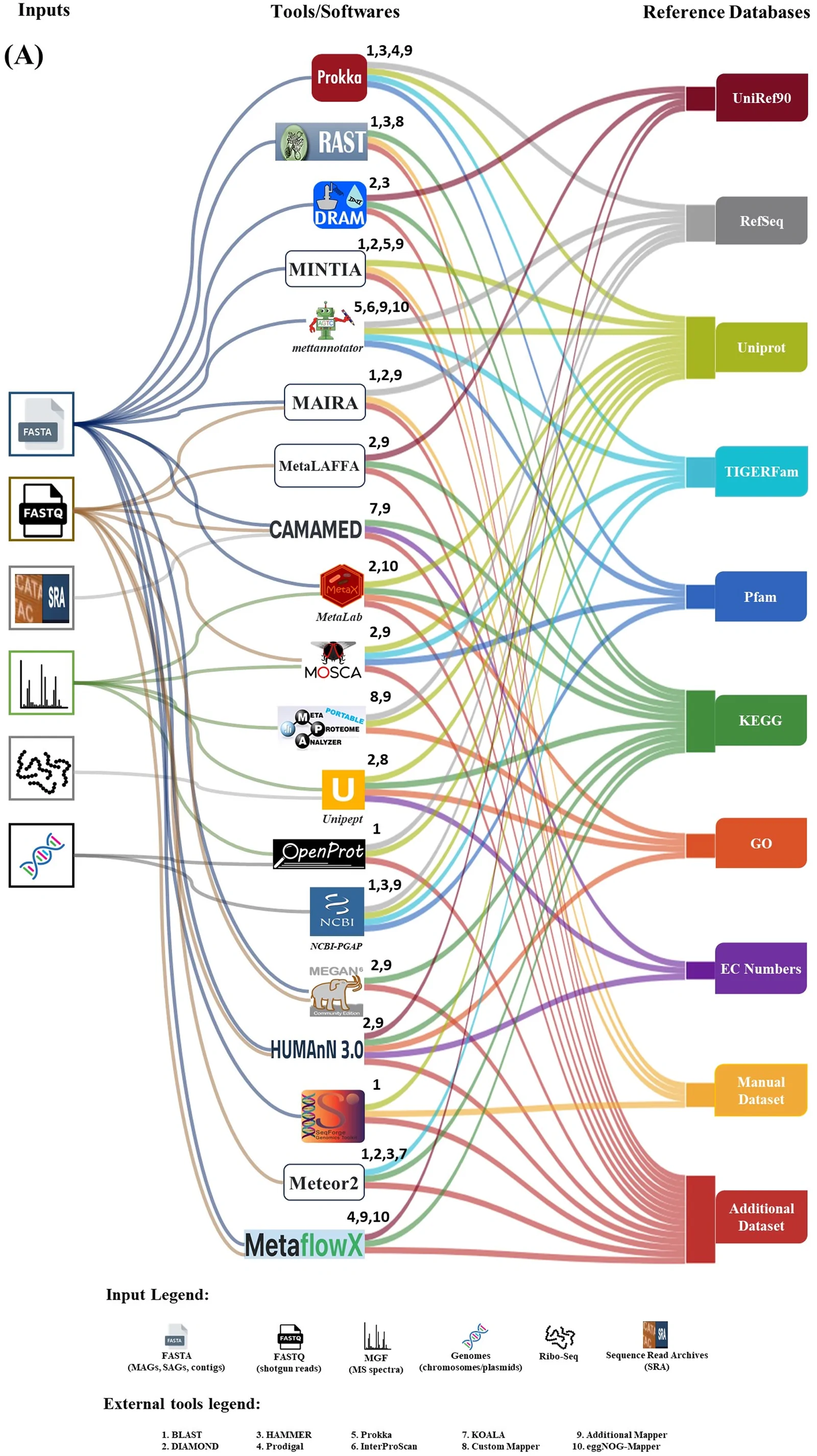

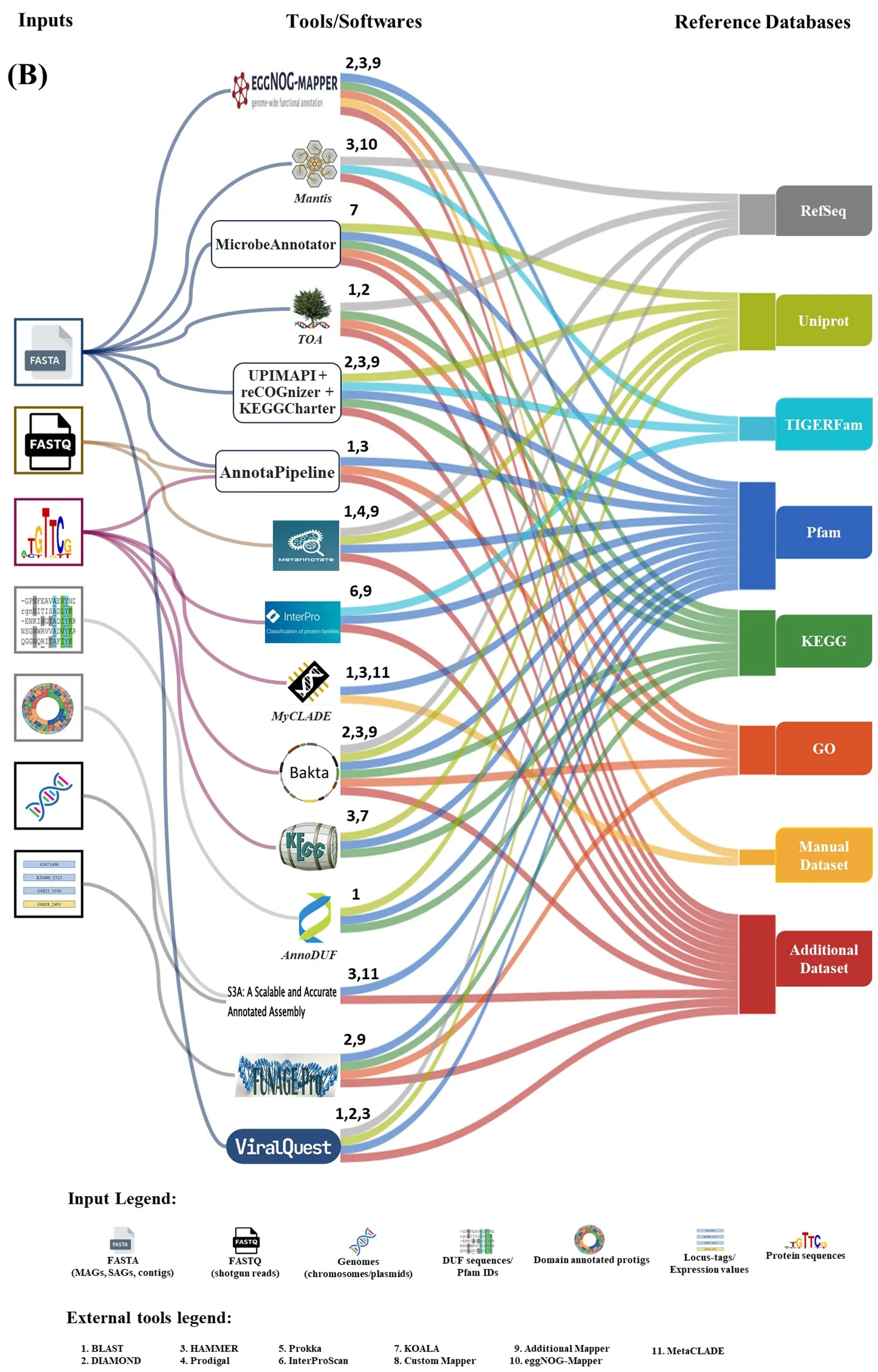

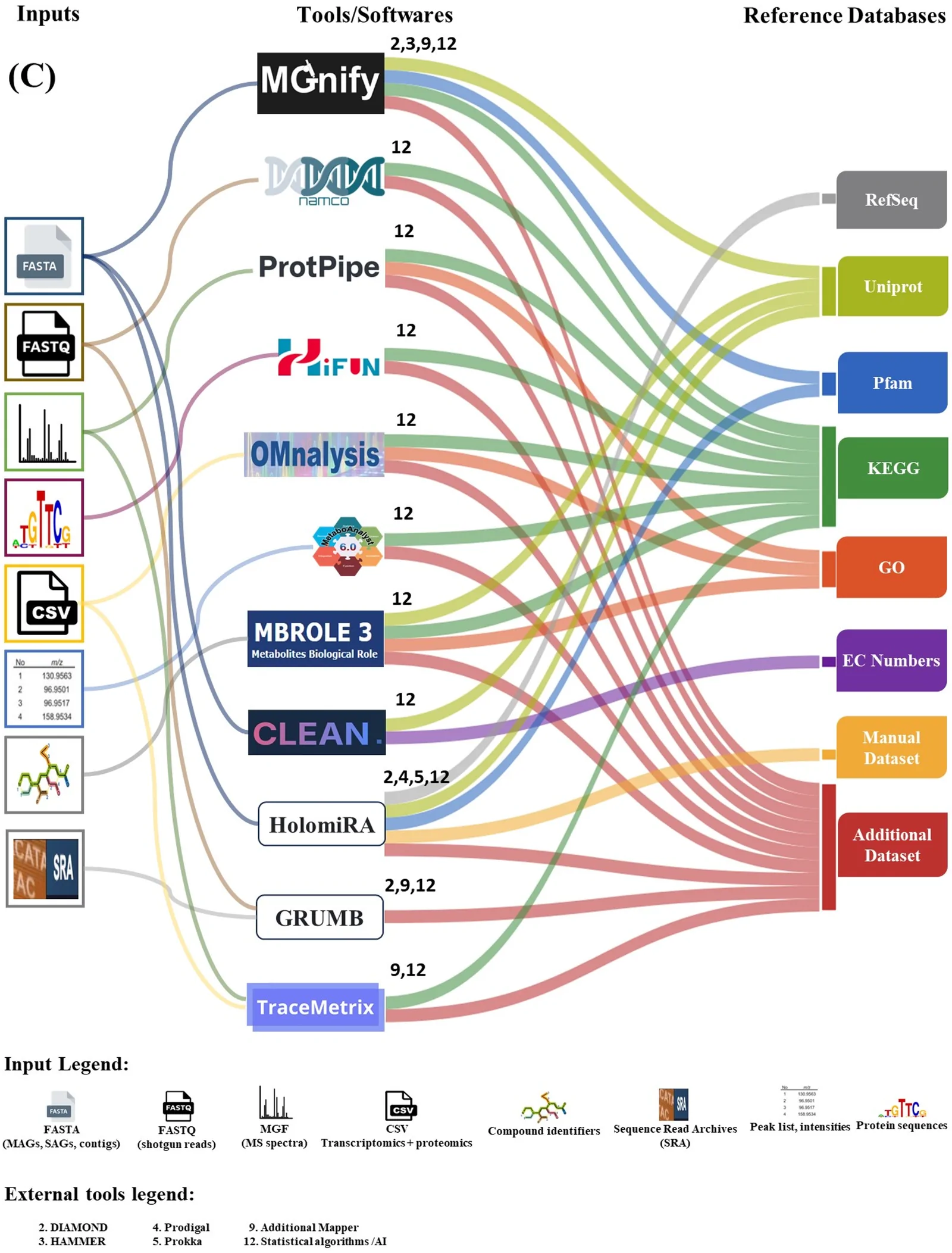
Map of the functional annotation ecosystem. Sankey diagrams depicting the relationships between input data types, annotation frameworks, integrated software dependencies, and reference databases. Tools are classified into three major categories according to their annotation strategy: homology-based (**A**), domain-centric (**B**), and statistical/AI-based (**C**) approaches. The left-hand nodes indicate accepted input formats, the central nodes represent annotation frameworks and associated software components, and the right-hand nodes correspond to the reference databases used for functional assignment. Connections highlight the resources and analytical dependencies that support functional annotation across different microbiome analysis workflows

Functional annotation in microbiome research is not a single method, but a family of inferential strategies that differ in the type of data they use, the reference knowledge they depend on, and the level of biological organization they reconstruct. To organize this landscape, we surveyed PubMed for 2020–2026 and identified 46 tools spanning homology-based, domain-centric, pathway-oriented, statistical, machine learning-driven, and integrative multi-omics approaches (Table 1, Supplementary S1). These tools differ not only in implementation, but also in the type, granularity, and interpretive depth of the biological inferences they generate. Figure 1 summarizes how representative tools connect input data types to annotation strategies, software dependencies, and reference databases.

**Table 1:** Functional annotation tools and pipelines used in microbiome research

SN	Tools/Pipelines	Input	Databases Used	Tools Used	Output	Latest Version	System Requirements
1.	Prokka [17]	Preassembled genomic DNA sequences in FASTA format	UniProtKB, RefSeq, Pfam, TIGRFAMs	Prodigal, RNAmmer, Aragorn, SignalP, Infernal, BLAST+, HMMER	Annotated genome with protein-coding genes, rRNA, tRNA, etc.	v1.14.5 [Nov 11, 2019]	~ 204 MB RAM per genome, Linear speedup with up to 8 CPU cores
2.	RAST [18]	Prokaryotic genome in the form of a set of contigs in FASTA format	SEED subsystems, FIGfams, KEGG	GLIMMER2, tRNAscan-SE, Custom FIGfam matcher, BLAST	Gene calls, gene functions, metabolic reconstructions	RASTtk [2015]	Processes 50–100 genomes/day, Distributed computing (Argonne National Lab)
3.	NCBI-PGAP [19]	Bacterial and archaeal genomes (chromosomes and plasmids)	RefSeq, NCBIFAMs, TIGRFAMs, Pfams, CDD (SPARCLE) [20], TCDB (transporters), VFDB (virulence factors) [21]	GeneMarkS-2+, tRNAscan-SE, Rfam [22], HMMER, BLAST	Annotated genome with protein-coding genes, tRNAs, sRNAs, etc.	v6.9 [Nov 18, 2024]	Require Linux, or some compatible container technology, CWL (Common Workflow Language), and about 30GB of supplemental data
4.	MEGAN6 [23]	BLAST output files, FASTQ/FASTA reads	NCBI-nr (non-redundant protein), NCBI-nt (nucleotide), KEGG, eggnog, InterPro2GO, SEED, GTDB, SILVA [24] (16 S/23S rRNA)	DIAMOND, MEGAN LCA algorithm, MinPath (pathway reconstruction), MeganServer (server-based data access, REST-API)	Interactive taxonomic tree, KEGG pathway maps, eggNOG/InterPro2GO/SEED annotations, read counts and relative abundances, PCoA plots, comparative heatmaps	MEGAN6 v6.25.10 [2024]	Windows, Unix/Linux, macOS; GUI requires display (X11 on HPC); memory requirements scale with dataset size (large datasets may require > 32 GB RAM)
5.	MetaProteomeAnalyzer (MPA) [25]	MGF spectrum files	UniProtKB, NCBI NR, custom DBs	X! Tandem, MS-GF+, PeptideProphet	Protein identifications with functional annotations (GO terms, pathways), Taxonomic assignments, Meta-protein groups (homologous proteins)	Server-v3.4 [Mar 24, 2020]	Tested on Windows and various Linux systems, preferably 4 GB at least, but highly recommended are 8 or 16 GB of RAM
6.	InterPro [26]	Protein sequences	Pfam, PROSITE, PRINTS, SMART, NCBIfam, SUPERFAMILY, CATH-Gene3D, PIRSF, PANTHER, HAMAP, CDD, SFLD	InterProScan, MaxQuant, Foldseek	Functional analysis, protein family classification, domain prediction	InterPro 104.0 [Feb 06, 2025]	High-performance computing (HPC) clusters recommended for large-scale analyses
7.	Unipept [27]	Individual peptides or complete metaproteome datasets	UniProtKB, Enzyme Commission, GO, KEGG	Lowest Common Ancestor (LCA) algorithm, DIAMOND	Functional analysis based on GO terms and EC numbers	Unipept 6.1.3 [Jan 24, 2025]	The Unipept web server runs on a virtual Debian machine with a dedicated 2.6 GHz Intel Xeon Hexa Core processor and 128 GB of RAM
8.	DRAM [28]	Assembly-derived FASTA (MAGs, SAGs, contigs)	KEGG, UniRef90, CAZy [29], MEROPS	DIAMOND, HMMER, DRAM distill	Catalog of microbial traits, metabolic annotations	DRAM 1.5.0 [Jan 03, 2024]	Requires 512 GB of RAM to build all databases if the UniRef90 is included, and at least 64 GB if it is skipped
9.	KEGG Mapper [30]	Protein-coding genes in the genome	PATHWAY, BRITE, KO, GENES, MODULE, DISEASE/DRUG	KOALA, SSEARCH, HMMER	KEGG molecular networks, pathway maps, BRITE hierarchies	v5.2 [July 01, 2024]	N/A
10.	MAIRA [31]	Files of sequences in FastA or FastQ format. It can be specified as a single file or a directory of input files	RefSeq, CARD [32] and VFDB	MinION, MinIT, LAST, DIAMOND, BLASTP	Interactive taxonomic and functional analysis of long read metagenomic sequencing data on a laptop, without requiring external resources	MAIRA [2020]	High-end laptop, ≥ 32 Gb of memory, 500G of SSD
11.	MetaLAFFA [33]	Raw FASTQ files (adapter-trimmed)	UniRef90 (2020), KEGG KO, KEGG module, KEGG pathway, hs37d5 human genome + 1000 Genomes decoy sequences	DIAMOND, MUSiCC, EMPANADA	Annotating shotgun metagenomic data with abundances of functional orthology groups	MetaLAFFA-1.0.1 [Nov 16, 2021]	Supported on Mac and Linux, but not on Windows, requires Conda (version 4.8 or greater) for installation and operation
12.	S3A [34]	Metagenomic reads (short/long reads), Domain-annotated protigs (amino acid sequences from ORFs)	HMMER (Pfam), MetaCLADE (custom domain library)	FragGeneScan, BCALM, HMMER, MetaSim/FlowSim (simulated datasets), Minia/Xander (comparison tools)	Contigs (assembled coding regions), GFF files (domain annotations), Statistical metrics (precision/recall), Graphical plots (performance evaluation)	S3A [2020]	High-performance computing (Intel Xeon CPU, 128 GB RAM, multi-threading), Compatible with Illumina/454 sequencing data
13.	HUMAnN3 [35]	QC-filtered metagenome/metatranscriptome reads (FASTQ/FASTA)	ChocoPhlAn, UniRef90, UniRef50, MetaCyc, KEGG, GO, EC	MetaPhlAn ≥v3.0, Bowtie2 ≥ v2.1, DIAMOND, MinPath	Gene families abundance table (RPK/CPM, UniRef90 IDs, species-stratified); pathway abundance and coverage tables (MetaCyc); normalized tables (EC, KEGG KO, GO terms)	v3.9 [Feb 21, 2024]	Linux or macOS; ≥16 GB RAM; ≥10 GB disk (databases); full UniRef90 database ~ 20.7 GB; ChocoPhlAn database ~ 15 GB
14.	Bakta [36]	Protein sequences via --proteins in either GenBank (CDS features) or Fasta format	Rfam, Mob-suite, DoriC, AntiFam, UniProt, RefSeq, COG, KEGG, PHROG, AMRFinder, ISFinder, Pfam, VFDB	tRNAscan-SE, Aragorn, INFERNAL, PILER-CR, Pyrodigal, PyHMMER, Diamond, Blast+, AMRFinderPlus, pyCirclize	Rapid & standardized local annotation of bacterial genomes & plasmids. Each annotated feature in a standardized machine-readable JSON file	v1.11 [Feb 26, 2025]	N/A
15.	CAMAMED [37]	FASTQ, FASTA or SRA file formats, in both paired-end and single-end modes	KEGG ortholog (KO) groups, Enzyme Commission (EC) numbers, and reactions, MetaPhlAn2 databases	SRA Toolkit, FastQC, KEGG Automatic Annotation Server (KAAS), GhostKOALA	Can analyze metagenome data at taxonomical and functional level, also it is capable of analyzing the potential functional differences at other functional levels, i.e., KO, EC number and reaction	CAMAMED [Jan 2021]	Software is available through Docker images at www.hub.docker.com
16.	eggNOG mapper [38]	FASTA files (nucleotide or protein sequences)	eggNOG v5 (Orthologous groups), COG, KEGG, Pfam, SMART, GO	Prodigal, DIAMOND, MMseqs2, or HMMER	Functional annotations, orthology assignments, domain predictions	v2.1.12 [Aug 18, 2023]	Storage: ~40 GB for eggNOG DBs, plus 9–86 GB depending on search mode Memory: Up to 44 GB RAM for annotation, ~ 2 GB per server with HMMER
17.	Mantis [39]	Protein sequence FASTA files	EggNOG, Pfam, KOfam, TIGRFAMs, NCBI Protein Family Models (NPFM)	HMMER, EggNOG-mapper, HMM	Consensus-driven protein function annotations	v1.5.5 [July 02, 2022]	Tested on Dell XPS 13-9370, 16GB RAM, Intel i7
18.	METAnnotatorX2 [40]	Both single-end, paired-end, and long reads data in .fastq format	Manually curated RefSeq NCBI (nucleotide), a RefSeq NCBI (amino acid), a custom Glycobiome database, a custom UniProt, and multiple host databases	samtools, prodigal, fastq-mcf, blast software suite, RapSearch2, SPAdes v3.14.0, CANU v2.0, Bowtie2 v2.4.0	Perform taxonomic and a range of functional profiling analyses of next-generation shotgun sequencing data	METAnnotatorX2 v1.0 [Oct 30, 2020]	The user needs to have at least 1 TB of free disk space to download all the requested databases
19.	MicrobeAnnotator [41]	Protein sequences in FASTA format	KEGG, UniProt (SwissProt, trEMBL), NCBI RefSeq, Pfam, InterPro, GO	KOfamScan	Functional annotation, KEGG module completeness matrix, Heatmap and barplot for metabolic reconstruction	v2.0.5 [April 27, 2021]	~ 19.4 GB RAM per genome
20.	MINTIA [42]	FastA files for genes and protein	NR: non-redundant protein database indexed for Diamond, Uniprot/Swissprot, COGs: database of Clusters of Orthologous Groups of proteins	Prokka, diamond, xvfb-run, MEGAN, rpsblast, samtools, tabix	HTML report includes a dynamic graphic with contig length and coverage for each sample	MINTIA [2021]	N/A
21.	MyCLADE [43]	List of protein sequences and possibly a target set of domains/clans	Pfam and QuickGO	MetaCLADE, PSI-Blast, HMMer, SCMs and CCMs models, Naive Bayes classifier	Targeted functional profiling of genomic and metagenomic sequences based on a database of a few million probabilistic models of Pfam domains	MyCLADE [2021]	The analysis of large files are not possible with MyCLADE, User can locally install the new improved version of MetaCLADE, MetaCLADE v2
22.	OMnalysis [44]	CSV file for transcriptomics data, xlsx file for the label free quantitative proteomics data	GO, KEGG, Reactome, PANTHER, biocarta, NCI-Nature Pathway Interaction Database PharmGKB and STRINGdb	clusterProfiler version 3.18.1, ReactomePA version 1.34.0, STRINGdb version 2.2.2	Option to download the converted accession IDs, GO enrichment and pathway enrichment analysis in CSV file format. All the diagrams produced in PCA, Plots, Statistical filtering, and GO heatmaps sections can be downloaded in TIFF, PNG, JPEG, and PDF format	OMnalysis [Nov 2021]	Minimum 4 GB of RAM, i3 processor (or equivalent). Compatible with any operating system (windows, Linux or Mac)
23.	TOA [45]	Genomic/transcriptomic sequences in FASTA format	Gymno 1.0, Dicots 4.0, Monocots 4.0, RefSeq Plant, NT (Nucleotide), NR (Non-Redundant Protein), GO, InterPro, KEGG, EC, MapMan, MetaCyc	BLAST+, DIAMOND, TransDecoder, SQLite	Functional annotations (GO terms, InterPro domains, KEGG pathways, EC numbers, MapMan BINs, MetaCyc), Alignment files (XML), Statistics, CSV reports, plots (e.g., GO term distributions)	v 0.66 [2021]	Recommended 32-core CPU, 256 GB RAM, SSD/HDD storage. Cloud-compatible via AWS (NGScloud2)
24.	AnnotaPipeline [46]	Based on FASTA (i) nucleotide or (ii) protein sequences or (iii) structural annotation files (GFF3), users can input FASTQ RNA-seq data, MS/MS data from mzXML or similar formats	SwissProt, Pfam, CDD, TrEMBL, EuPathDB, NCBI | NR	BLAST + and RPS-BLAST, InterProScan, HMMER	The pipeline uses both transcriptomic and proteomic information to corroborate annotations and validate gene prediction, providing transcription and expression evidence for functional annotation	AnnotaPipeline [Nov 2022]	Performance tests were carried out using a computational cluster equipped with 40 threads processor (3.2 GHz), 285 GB RAM memory (DDR4, 2,400 MHz), and 5 TB storage space (2.5 SATA HD, 7,200 RPM)
25.	Namco [47]	FASTQ files, OTU/ASV tables	SILVA (taxonomy), PICRUSt2 (functional inference), KEGG (via PICRUSt2)	DADA2, LotuS2, phyloseq, vegan, NetCoMi	Taxonomic profiles, diversity metrics, co-occurrence networks, KEGG maps	Namco [2022]	N/A
26.	FUNAGE-Pro [48]	Single list of locus-tags (equal weight), Single list with values (e.g., fold change), Multiple experiments (e.g., time-series), Clusters or Modules (e.g., k-means, gene networks with clusterID)	Complete bacterial genomes from RefSeq and GenBank (> 50,000 genomes), annotated using GO, COG, EggNOG-COG, EggNOG, InterPro, KEGG, Pfam, KEYWORDS, OPERONS	DIAMOND, R/Bioconductor (hypergeometric test, Benjamini-Hochberg correction), Genome2D	Summary Table (overrepresented ClassIDs per class and experiment/cluster), Main Table (ClassID, description, score, hits, p-value, gene set), Bar Graphs (Bar 1: number of ClassIDs per experiment/cluster, Bar 2: p-values of ClassIDs in –log scale, Bar 3: hits for selected ClassID across experiments)	FUNAGE-Pro [April 2025]	N/A
27.	UPIMAPI + reCOGnizer + KEGGCharter [49]	Protein sequences (FASTA)	UniProtKB, KEGG, BioCyc, CDD, eggNOG, COG, KOG [50], Pfam, SMART, TIGRFAM, NCBIfam	DIAMOND, UniProt API, BioPython, RPS-BLAST, HMMER, rpsbproc, blast-formatter	Functional annotations (EC numbers, KEGG IDs, GO terms, taxonomic lineage)	v1.6.4 [2022]	Tested on a #116-Ubuntu SMP kernel, with 126 GB of memory and 16 threads
28.	HIFUN [51]	Protein sequences	UniProtKB, Molecular Function Ontology (MFOs), UHGP-50 catalogue	CD-Hit, CAFA3	Homology Independent Protein Function prediction on Protein-Language Self-Attention Model (deep learning architecture)	HiFun [2023]	GPU-accelerated (TensorFlow/Keras)
29.	MBROLE3 [52]	Chemical compound identifiers (KEGG, HMDB, YMDB, ECMDB, PubChem, ChEBI, LMSD, CAS, ChemSpider, MeSH, PathBank, PharmGKB, Reactome, TTD)	KEGG, BioCyc, HMDB, PathBank, Reactome, PharmGKB, CTD (Gene Ontology), ChEBI, LMSD, TTD, LIPID MAPS, ECMDB, YMDB, MeSH, UniProt, PANTHER, PubMed (indirect annotations)	Statistical enrichment algorithms, ID conversion utilities	Interactive tables (enriched annotations), Graphical plots (bar/dot plots)	MBROLE3 [2023]	N/A
30.	Meta4P [53]	Protein FASTA files	eggNOG database, KEGG orthology, KEGG pathways, KEGG modules, KEGG reactions	eggNOG-mapper	Integrate label-free quantitative metaproteomic data with taxonomic and functional annotations	Meta4P 1.5.5 (LTS) [Feb 27, 2024]	Tested on a Windows computer (Processor Intel Core i7-5500U CPU @ 2.40 GHz 2.40 GHz, 8GB RAM)
31.	MetaLab-MAG [54]	MS/MS raw files, Protein/peptide sequences (FASTA)	MAGs Databases: UHGG v2.0 (human gut), Human Oral v1.0, Cow Rumen v1.0, Marine v1.0 Functional annotation: eggnog, GO, KEGG, GTDB (Genome Taxonomy Database r202), UniProt	pFind, DIAMOND, eggNOG-mapper (emapper), featureCounts, minimap2	Annotated tables (peptides, proteins, genomes, taxa, functions), Functional annotations (GO terms, KEGG pathways, eggNOG categories), Web-based visualization reports (PCA, heatmaps, etc.)	MetaX [2024]	Requires 8 GB RAM, Quad-Core processors (3.0 GHz), 32.6 GB disk space
32.	MGnify [55]	Microbiome-derived nucleic acid sequences (metabarcoding, metatranscriptomic, metagenomic datasets), Long-read sequencing data (PacBio, Oxford Nanopore), Hybrid sequencing datasets (long + short reads), Sample metadata from publications and CDCH	MGnify protein database (MGYP accessions), Pfam, KEGG, Genome Properties, ENA, INSDC, UniProt	Prodigal, FragGeneScan, HMMER, MMseqs2, antiSMASH, PRICE algorithm, COBS, Sourmash, MySQL, ProtENN2 (deep learning annotations), MGnifyR, SIAMCAT (machine learning workflows)	Functional annotations (Pfam, KEGG), Metagenome-assembled genomes (MAGs), Non-redundant protein sequences (MGYP accessions), Pathway predictions, Biosynthetic gene clusters (BGCs), Genomic context data (MGYC contigs), Downloadable data (TSV, FASTA), Jupyter notebooks for downstream analysis	MGnify [2023]	Web Interface: Accessible via browsers (Chrome, Firefox, Safari, Edge)
33.	AnnoDUF [56]	DUF sequences or Pfam IDs (retrieved from Pfam database)	Pfam (Pfam-A.fasta), UniProt, KEGG	BioPython (SeqIO), Multiprocessing, PSI-BLAST	Annotating Functions of Proteins Having Domains of Unknown Function	AnnoDUF [2024]	N/A
34.	MetaboAnalyst [57]	Peak list (m/z, p-values, t-scores), Peak intensity table (generic, MZmine), Retention time (optional)	Pathway libraries (26 organisms: human, mouse, zebrafish, C. elegans, etc.), ~ 9,000 metabolite sets (disease-associated, chemical classes), KEGG, HMDB	Modified Gene Set Enrichment Analysis (GSEA), Mummichog algorithm (RT-enhanced)	Pathway enrichment results, Interactive heatmaps, Empirical compound annotations, Network exploration, Downloadable reports (tables, images, PDF)	MetaboAnalyst 6.0 [2024]	Web Interface: Accessible via browsers (Chrome, Firefox, Safari)
35.	MOSCA [58]	Contigs or preprocessed reads (assembled or not)	UniProt, CDD, Protein Clusters, TIGRFAM, NCBIfam, Pfam, SMART, COG, KOG	Snakemake, FragGeneScan, UPIMAPI, reCOGnizer	Can analyze and interpret meta-omics data, including metagenomics, metatranscriptomics, and metaproteomics	v2.3.0 [Jan 30, 2024]	N/A
36.	OpenProt [59]	Eukaryotic genome annotations (NCBI RefSeq, Ensembl), Ribosome profiling (RIBO-seq) datasets, Mass spectrometry (MS) proteomics datasets	NCBI RefSeq, Ensembl, UniProt	PRICE algorithm (ribosome profiling analysis), SearchGUI/PeptideShaker (MS data processing), BLAST (isoform identification), PostgreSQL (database management), Flask (web interface framework)	Predicted ORFs categorized as RefProts/novel isoforms/AltProts, Evidence from ribosome profiling/MS/conservation/functional domains, Protein isoform relationships, Downloadable data (TSV/FASTA/BED formats)	OpenProt v1.6 [2021]	Web Interface: Accessible via browsers (Chrome, Firefox, Safari, Edge)
37.	mettannotator [60]	Prokaryotic genome assemblies (FASTA), Comma-separated text file (prefix, assembly path, NCBI Taxid), Supports isolate/MAGs (bacterial/archaeal)	UniProt (UniRule, ARBA), InterPro, eggNOG, KEGG, ChEBI, AntiFam, PGAP, Custom databases (biosynthetic gene clusters, antimicrobial resistance, etc.)	Prokka (default), Bakta (bacteria only), Pseudofinder, InterProScan, eggNOG-mapper, UniFIRE, SanntiS (biosynthetic clusters), CRISPRCasFinder, tRNAscan-SE	GFF file (merged annotations), Circos plots (PyCirclize), Functional predictions (genes, ncRNA, pseudogenes), Biosynthetic clusters, AMR genes, Anti-phage systems	v1.4.0 [2025]	Nextflow, Singularity/Apptainer/Docker, 12 GB RAM, 8 CPUs, 182 GB disk space (databases), Linux/macOS
38.	ProtPipe [61]	Raw MS data (DIA/DDA formats), Protein/peptide abundance files from DIA-NN, Spectronaut, or FragPipe	GO, KEGG pathways, STRING (protein-protein interactions), UniProt	DIA-NN, Spectronaut, FragPipe, clusterProfiler (GO/KEGG), STRING-DB (PPIs), MHCflurry (MHC-peptide binding)	Pathway enrichment, PPI networks, MHC binding affinity predictions, Heatmaps, PCA/UMAP plots	ProtPipe [2025]	10 CPUs, 12 GB RAM (e.g., 10 samples take ~ 5–13 h). Scalable to HPC for large datasets
39.	MetaflowX [62]	Paired-end or single-end FASTQ, pre-assembled contig FASTA	eggNOG v5 (COG), KEGG, CAZy, CARD v3.2.7, VFDB, MetaPhlAn4, Kraken2, GTDB r226, UniRef90	Fastp, Trimmomatic, Bowtie2, MetaPhlAn4, Kraken2, HUMAnN3, metaSPAdes, MEGAHIT, Prodigal, CD-HIT, eggNOG-mapper	QC reports, taxonomic & functional profiles, non-redundant gene catalog, HQ MAGs	MetaflowX [2025]	Nextflow, ~ 436.6 GB database storage, tested on 32-core, 64 GB RAM, compatible with AWS, Tencent Cloud, and HPC
40.	Meteor2 [63]	Paired-end FASTQ reads	KEGG, CAZy, ResFinder + ResFinderFG, GTDB r220, eggNOG 3.0, TIGRFAM 15.0, GBMs, GMMs	Bowtie2 v2.5.4, Freebayes v1.3.8, MSPminer v1.1.3, KofamScan v1.3.0, dbCAN3 v4.1.4, DIAMOND v0.9.22, hmmsearch v3.2.1, blastn v2.15.0/2.16.0	Gene count tables, MSP abundance profiles, KEGG/GMM/GBM functional module completeness, CAZyme profiles, ARG profiles, strain-level phylogenetic trees	v2.0.18 [Nov 2025]	Python ≥ 3.10, ~ 5 GB RAM in fast mode, compatible with HPC and local workstations
41.	SeqForge [64]	Individual FASTA files, directories of FASTA files	User-defined BLAST+ databases, NCBI GenBank/JSON, MIBiG (biosynthetic gene clusters), UniProt	BLAST + 2.16.0 (blastn, blastp, tblastn)	Concatenated BLAST results tables, standard BLAST+ alignment files, heatmaps, extracted FASTA sequences (seqforge extract), extracted full contigs, metadata tables, assembly statistics	SeqForge [2025]	Python ≥ 3.10, tested on Linux Ubuntu v24.04 and macOS v12.7.6
42.	TraceMetrix [65]	LC–MS raw data, metabolite abundance matrices (CSV)	Blood Exposome, KEGG, T3DB, DrugBank, HMDB, MassBank, MoNA, Metabolomics Standards Initiative (MSI)	ProteoWizard MSConvert, ThermoRawFileParser, XCMS, Spring Boot, Vue + ElementUI	Annotated metabolite feature tables (MS1/MS2); PCA, PLS-DA, OPLS-DA results, KEGG pathway enrichment, interactive HTML visualizations	TraceMetrix [2025]	Web-based (Chrome, Safari), deployed on HPC cluster (CentOS 7.9.2009, Slurm)
43.	GRUMB [66]	SRA accessions, paired-end FASTQ files	CARD (AMR), VFDB (virulence), Kraken2/Bracken species database, GTDB (taxonomy)	FastQC, BBMap/BBTools, metaSPAdes, MetaBAT2, CheckM2, Kraken2 + Bracken, DIAMOND	Assembled MAGs, taxonomic profiles, AMR/virulence hit tables, species-abundance matrices, ML risk scores [0,1]	GRUMB [Sept 2025]	Linux (Ubuntu 20.04+), Python 3.11, SLURM-compatible HPC
44.	HolomiRA [67]	Prokaryotic genome sequences (FASTA), mature host miRNA sequences (FASTA)	MirGeneDB, miRBase, miRDB, RefSeq, UniProt, Pfam, SUPER-FOCUS functional database	Snakemake ≥ 7.32.3, Prokka v1.14.6, Prodigal v2.6.3, RNAhybrid v2.1.2, RNAup v2.5.1, SUPER-FOCUS v1.4.1, DIAMOND v2.1.8	Predicted miRNA–prokaryotic gene binding sites (TSV), summary tables, bar plots, Venn diagrams, SUPER-FOCUS enrichment tables (Level 1–3), differential abundance heatmaps	HolomiRA [Oct 2025]	Linux (tested); Snakemake ≥ 7.32.3, all dependencies auto-installed via Conda/Mamba, no specific RAM/CPU requirements
45.	ViralQuest [68]	Pre-assembled contigs in FASTA format	NCBI Viral RefSeq, NCBI nr, RVDB-prot v29.8, Vfam release 228, eggNOG v4.5 viral NOGs, Pfam	ORFiPy, Diamond BLASTx v2.1.9+, BLASTn, pyHMMER v3.3.2	Interactive HTML report: taxonomy dashboard, ORF maps, BLASTx/BLASTn results, ICTV lineage, Pfam domains, AI narrative, composite confidence score [0–100]	ViralQuest [2026]	GNU/Linux, Python ≥v3.12, Docker ≥v28.1.1, GPU optional (local LLM via Ollama)
46.	PICRUSt2 [69]	Amplicon sequence variants (ASVs)/OTUs in FASTA, abundance/feature table (BIOM/TSV)	PICRUSt2-SC reference database (curated bacterial + archaeal reference genomes/phylogenies), KEGG Orthologs, EC numbers, MetaCyc, COG, Pfam, TIGRFAM	HMMER, castor algorithm, MinPath, gappa	Predicted gene-family abundance tables, predicted MetaCyc pathway abundances	v2.6.3 [Nov 2025]	N/A

The table summarizes the primary input data accepted by each tool, the reference databases and auxiliary software employed, the type of functional output generated, the most recent available version at the time of review, and the corresponding computational requirements. N/A, not available

Homology-based annotation remains the dominant strategy for assigning biological function to microbial genes and proteins. These approaches infer putative functions by transferring annotations from experimentally characterized sequences to homologous query sequences identified through primary sequence similarity searches using tools such as BLAST [70], DIAMOND [71], HMMER [72], or MMseqs2 [73]. Widely used reference databases include UniProtKB [74] and RefSeq [75], which provide curated protein sequences, standardized annotations, and extensive cross-references to complementary resources such as KEGG [76], InterPro [26], and BioCyc [77], and Gene Ontology (GO) [78]. Genome annotation pipelines such as RAST [18], Prokka [17], Bakta [36], and NCBI-PGAP [19] integrate gene prediction, similarity searches, and functional assignment into scalable workflows applicable to bacterial and archaeal genomes as well as metagenomic assemblies. Homology-based frameworks primarily infer encoded functional capacity, providing information about the metabolic and biochemical potential that may be present within microbial genomes or communities. Their main limitation is that performance depends strongly on reference database completeness and sequence conservation, which reduces annotation accuracy in phylogenetically novel or poorly characterized systems. It is also worth noting that sequence homology can support inference of broad enzymatic function while remaining insufficient to resolve substrate specificity, particularly among related enzymes with overlapping or promiscuous activities [79, 80]. To improve annotation sensitivity for divergent proteins and incomplete homologs, many frameworks focus on conserved domains, motifs, and orthologous groups rather than full-length sequence similarity. Tools such as InterProScan [26] and eggNOG-mapper [38] integrate databases including Pfam [81], SMART [82], TIGRFAMs [83] and COGs [84] to identify conserved structural or evolutionary signatures associated with specific functional properties [85]. These approaches enable partial functional inference even when no strong full-length homolog is available. By identifying catalytic motifs, structural folds, or conserved protein families, domain-centric systems extend annotation into more divergent regions of sequence space and improve coverage across environmental microbiomes. Specialized frameworks such as MyCLADE [43] and AnnoDUF [56] further target domains of unknown function (DUFs), attempting to refine poorly characterized protein families through structural, phylogenetic, or contextual evidence. Compared with classical homology transfer, domain-centric approaches often provide broader sensitivity across divergent taxa, although they typically infer partial biochemical properties rather than fully resolved organism-level functions. An additional source of uncertainty is that genome annotation and subsequent functional inference do not operate equivalently across the biological domains represented within microbiomes. Many widely used genome-annotation pipelines have been developed specifically for bacteria and archaea, including RAST [18], Prokka [17], Bakta [36], and NCBI-PGAP [19]. In microbial eukaryotes, by contrast, functional annotation additionally depends on accurate reconstruction of eukaryotic gene structures, such that uncertainty in exon–intron boundaries and coding-sequence prediction can propagate into downstream functional assignments. Eukaryote-oriented frameworks such as BRAKER3 [86] and GeneMark-ETP [87] therefore integrate genomic sequence with transcriptomic and/or protein evidence to improve protein-coding gene prediction. Viruses, although outside the cellular domains of life, present a distinct challenge because extensive sequence divergence can limit homology-based functional assignment [88]. Thus, the confidence associated with a functional annotation depends not only on the annotation strategy and reference resource used, but also on the biological domain being investigated.

Functional annotations are frequently contextualized within higher-order biochemical systems through pathway reconstruction and metabolic network mapping. Databases such as KEGG, MetaCyc, and BioCyc organize genes and proteins into structured representations of metabolic pathways, enzymatic modules, and cellular processes. Tools including KEGG Mapper, DRAM and MetaLab-MAG [54] use these resources to reconstruct metabolic pathways from genomic, metagenomic, or metaproteomic data. These frameworks move functional interpretation beyond individual genes by inferring pathway completeness, metabolic capabilities, and potential ecological roles. In community-scale microbiome studies, pathway-based approaches estimate the collective metabolic potential of microbial consortia by aggregating annotations across taxa. This enables the identification of processes such as carbon utilization, nitrogen cycling, or stress-response pathways at the ecosystem level [89]. Pathway-oriented systems therefore provide an intermediate layer of inference between individual molecular functions and higher-order ecological organization. However, inferred pathways do not necessarily correspond to coordinated biological activity, particularly in metagenomic datasets where pathway components may be distributed across multiple community members. Functional capacity can also be predicted indirectly from metabarcoding data [2]. Approaches such as PICRUSt2 [69] use marker-gene sequences and phylogenetic placement relative to organisms with characterized genomes to infer the gene families and pathways potentially represented within a community. Unlike shotgun metagenomics, however, metabarcoding does not directly observe the genes on which these functional predictions are based. The resulting profiles therefore introduce an additional layer of inference between community composition and functional capacity and depend on the availability and phylogenetic proximity of suitable reference genomes, as well as on the extent to which gene content is conserved among related organisms. Strain-level variation, gene gain and loss, and horizontal gene transfer can consequently decouple marker-gene relatedness from functional repertoire. Metabarcoding-derived functional profiles should therefore be interpreted as predictions of community functional capacity rather than direct measurements of encoded functions, and do not by themselves provide evidence of functional realization or impact.

Recent annotation strategies increasingly integrate statistical learning and machine learning approaches to address limitations associated with classical homology-based inference. These methods leverage latent sequence patterns, protein embeddings, structural predictions, or evolutionary context to improve functional prediction for divergent or previously uncharacterized proteins. Structure prediction has become particularly influential following AlphaFold2 [90], which demonstrated that protein structures can be predicted from amino-acid sequence with high accuracy across a broad range of proteins. Predicted structures can support functional inference by revealing structural relationships, conserved folds, or putative functional sites that may not be apparent from primary sequence similarity alone. However, structural prediction does not itself establish biochemical function, and structure-based assignments therefore remain inferential and benefit from integration with sequence, genomic-context, and experimental evidence. Representative examples include CLEAN [91], HIFUN [51], and related AI-assisted frameworks trained on protein families, domain architectures, or Gene Ontology annotations. Other platforms such as MAIRA [31] and MINTIA [42] combine sequencing-based analyses with real-time taxonomic and functional profiling, while enrichment-oriented tools such as FUNAGE-Pro [48] and MBROLE3 [52] integrate transcriptomic, proteomic, and metabolomic datasets to identify overrepresented functional categories across multiple omics layers.

Machine learning-driven approaches expand the observable functional landscape by extending annotation beyond direct sequence similarity and improving sensitivity across poorly represented protein families. At the same time, these methods remain dependent on training data derived from experimentally characterized proteins and therefore inherit many of the biases and limitations embedded within current reference knowledge.

Increasingly, microbiome functional analysis relies on integrative platforms capable of combining genomic, transcriptomic, proteomic, and ecological information within unified analytical frameworks. Workflows such as AnnotaPipeline [46] and OpenProt [59] integrate homology searches with ribosome profiling and mass spectrometry to refine gene boundaries, identify novel isoforms, and improve functional interpretation of coding sequences. Large-scale modular pipelines including MetaLAFFA [33] and MOSCA [58] combine sequence alignment (e.g., DIAMOND), clustering (e.g., UniRef90), pathway reconstruction (e.g., KEGG), and abundance profiling into scalable metagenomic workflows, while community-oriented resources such as MGnify [55] and CAMAMED [37] aggregate annotations across microbial taxa to generate ecosystem-level functional profiles. Specialized platforms further extend annotation toward specific applications, including antimicrobial resistance detection [60], host-associated microbiomes [40, 60], and plant-associated [45] microbial systems. Together, these integrative frameworks expand the range of microbial functions that can be inferred, but they do so at different levels of resolution and with different forms of uncertainty. Understanding what each framework actually infers is essential for interpreting the biological meaning of microbiome functional profiles.

## What functional annotations actually mean

**Fig. 2:**
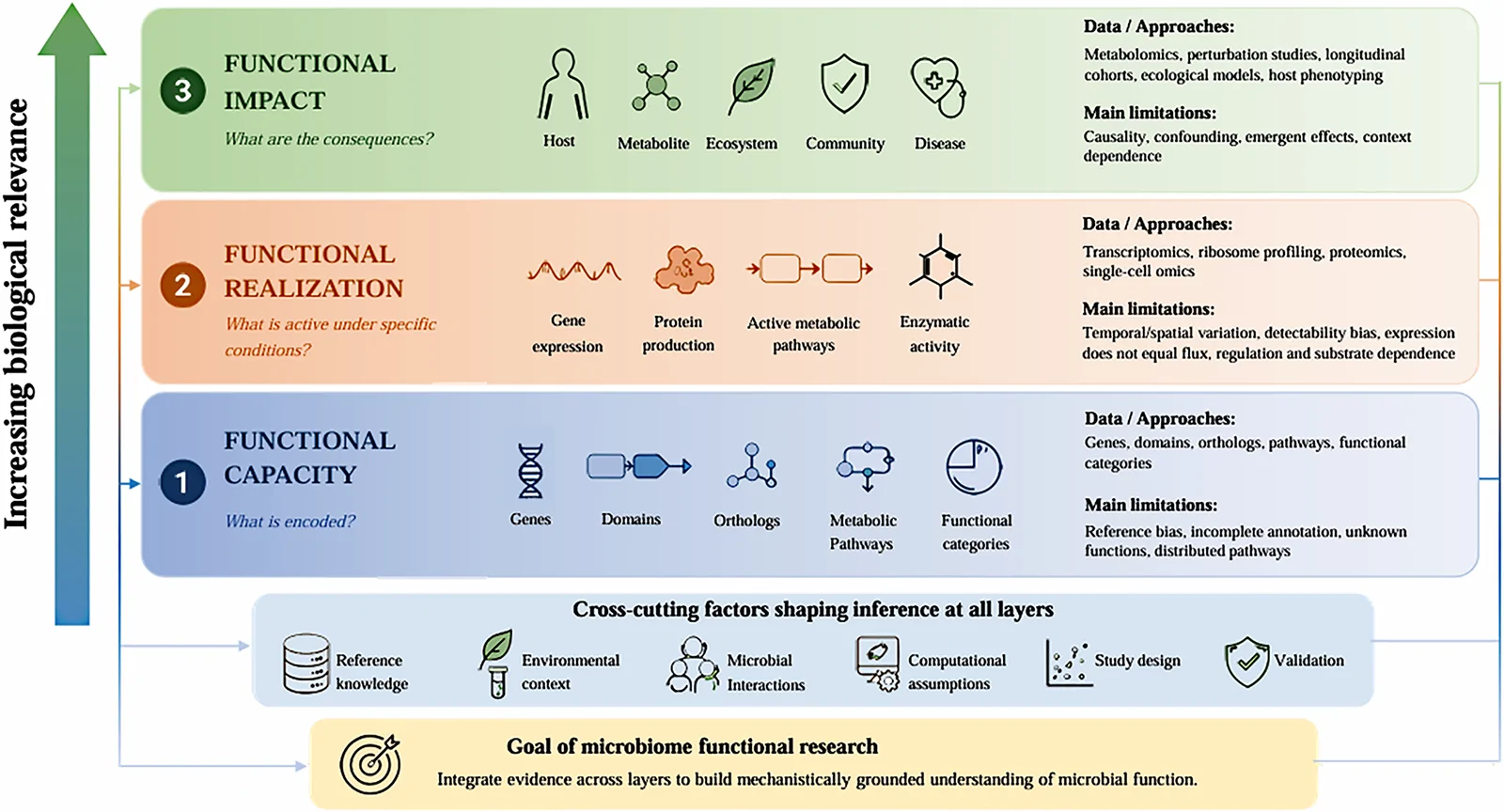
Layers of functional inference in microbiome research. Microbiome function is inferred at multiple, non-equivalent levels of biological evidence. Functional capacity captures what is encoded in the microbiome, as inferred from genes, domains, orthologs, and pathway annotations. Functional realization captures which molecular functions are actively engaged under specific conditions, based on metatranscriptomic, metaproteomic, and activity-centered measurements. Functional impact captures the resulting consequences for hosts, microbial communities, or ecosystems, as assessed through metabolomics, perturbation studies, longitudinal analyses, ecological modeling, and host phenotyping. Cross-cutting factors shape inference at all levels, including reference knowledge and database completeness, environmental context, microbial interactions and spatial structure, computational assumptions, study design, and experimental validation. Together, these layers illustrate that microbiome “function” is not a single measurable property, but a hierarchy of inferences. Each layer is supported by different types of evidence and is subject to distinct sources of uncertainty.

Functional annotations support different kinds of functional inference, but these inferences do not all describe the same biological level. Some approaches primarily support inference of functional capacity, others provide evidence of functional realization under particular conditions, and still others aim to establish functional impact on hosts, microbial communities, or ecosystems (Fig. 2). Distinguishing among these layers is essential for interpreting microbiome function correctly.

Sequence similarity, conserved domains, and ortholog-based annotations primarily support claims about functional capacity, the genes, proteins, and pathways that are encoded in a genome or community of organisms. Homology-based annotation is strongest when close reference matches exist, whereas domain-centric methods can extend inference into more divergent sequence space by recognizing conserved catalytic or structural signatures. Pathway reconstruction further organizes these annotations into higher-order metabolic systems, but the resulting pathways describe inferred potential rather than direct activity.

Transcriptomics and proteomics of single organisms, metatranscriptomics and metaproteomics of microbial communities, and related activity-centered methods move interpretation closer to functional realization. These approaches provide evidence that specific genes, proteins, or pathways are engaged under particular conditions. Even here, however, measured expression or protein abundance does not by itself establish physiological importance, because realized activity depends on regulation, abundance thresholds, spatial context, availability of substrates, and environmental constraints. However, because living systems are inherently resource-efficient rather than wasteful, protein abundance is generally considered a reliable proxy for activity level [92, 93].

Functional impact refers to the measurable consequences of microbial activity for hosts, communities, or ecosystems. Such impact may be observed through host phenotypes, metabolite profiles, perturbation responses, ecological restructuring, or experimentally validated metabolic fluxes. Unlike capacity or realization, impact is not a property that can be read directly from annotation alone; it emerges from the interaction between microbial activities and their biological context.

These levels of evidence are related but not equivalent. Capacity does not imply activity, activity does not imply consequence, and consequence may arise from interactions that are invisible to gene-centered annotation [94, 95]. Likewise, reconstructed pathways may reflect distributed community potential rather than coordinated organism-level metabolism, and expression profiles may indicate engagement without demonstrating ecological relevance. If these distinctions are not made explicit, functional annotation can be overinterpreted as direct evidence of microbial behavior. Treating inferred capacity, realized activity, and biological impact as interchangeable obscures uncertainty, weakens mechanistic claims, and limits comparability across studies. Functional profiles are therefore best interpreted as layered models of inference rather than direct observations of function itself.

## The unevenly observable functional landscape

The functional landscape visible to annotation is highly uneven across microbiomes, with well-studied host-associated systems far more legible than environmentally diverse or phylogenetically underrepresented ecosystems [96]. This uneven observability also extends across biological domains [86], because differences in gene-prediction requirements and the depth of available reference knowledge influence which components of complex microbiomes can be functionally resolved. In the Unified Human Gastrointestinal Protein (UHGP) catalog, combining eggNOG-mapper with InterProScan, COG, and KEGG enabled annotation of approximately 60% of more than 170 million protein sequences, leaving nearly 40% without functional assignment [97]. By contrast, well-characterized model organisms such as *Escherichia coli* K-12 achieve annotation rates exceeding 80–90% across multiple annotation frameworks [41, 98]. Annotation performance declines further in phylogenetically novel systems. Candidate Phyla Radiation (CPR) genomes [99], often used as proxies for poorly represented environmental lineages, exhibit annotation rates of only ~ 60–65% across commonly used tools including DRAM, eggNOG-mapper, and MicrobeAnnotator [41]. Marine and soil microbiomes show similar limitations in functional resolution, with Tara Oceans OM-RGC.v2 catalog assigning only 23.6% of genes to KEGG orthologous groups [100], and the SMAG soil catalog classifying 78.4% of species-level genome bins as previously unknown species [101]. Microbiome functional profiles therefore reflect not only biological organization, but also the current boundaries of observable biochemical space.

This uneven visibility matters because unresolved sequence space is not simply residual noise. Unknown proteins and unassigned genes define the limits of what can be inferred, biasing functional interpretation toward better-characterized taxa, environments, and biochemical processes. In that sense, “functional dark matter” is not a minor gap in annotation coverage, but a structured region of biological space that remains only partially accessible to current frameworks [96].

Expanding observability is not limited to sequence annotation. Proteome-level approaches increasingly provide direct access to realized molecular activity within microbial communities [102]. Metaproteomics and related activity-centered methods reveal proteins synthesized under defined conditions and therefore extend interpretation beyond encoded potential. However, proteome observability is still constrained by peptide detectability, extraction efficiency, analytical depth, and database-dependent identification, such that substantial fractions of realized microbial activity remain only partially covered. Interestingly, the enhanced analytical depth achieved by the latest generation of high-resolution tandem mass spectrometers promises a more comprehensive view of metaproteomes [103], and consequently an enlarged panorama of inferred functions.

Incomplete observability does not preclude meaningful inference, but it does mean that microbiome function is always described from a partial view. Comparative and longitudinal studies can still recover coherent ecological, metabolic, and host-associated functional patterns even when large fractions of genes or proteins remain unresolved [12, 104]. The challenge is to interpret those patterns while explicitly acknowledging the unknown and incompletely visible portions of the system.

## Toward context-aware and mechanistically grounded microbiome function

If microbiome function is understood as a distinction among capacity, realization, and impact, then the next step for the field is not simply better annotation, but better inference. Functional profiles should be interpreted as context-dependent representations of what microbial communities may encode, what they actively do, and what consequences they produce under specific environmental or host conditions. This requires moving beyond additive pathway counting toward models that incorporate ecological interactions, spatial organization, and perturbation history [105–109].

Community coalescence studies, longitudinal analyses, and ecological network models already show that similar functional profiles can yield distinct behaviors depending on host physiology, temporal dynamics, and interspecies interactions [106, 109–112]. The implication is that functional organization should be treated as conditional rather than universal. Future studies should therefore report not only what functions are detected, but also the context in which they are interpreted and the level of inference they support.

A second priority is to integrate annotation with mechanistic models without collapsing distinct evidence layers. Genome-scale metabolic models (GEMs), constraint-based frameworks, and community metabolic reconstructions can embed annotated functions within chemically and physiologically constrained systems, making it possible to predict nutrient utilization, metabolic exchange, ecological competition, and responses to perturbation. Their value, however, depends on experimental validation. Predictions from such models should be tested using metabolomics, perturbation experiments, isotope tracing, or activity-centered assays, rather than treated as equivalent to direct observation.

Activity-centered methods such as metaproteomics help bridge the gap between encoded capacity and realized biochemical activity [10]. These approaches should be used not as replacements for annotation, but as evidence that can connect annotated potential to function under defined conditions. In parallel, advances in machine learning and structure-aware annotation can extend inference into poorly characterized sequence space by improving sensitivity for proteins lacking strong homology to known references. These methods are most useful when treated as complements to classical annotation rather than substitutes for context-rich biological evidence.

Finally, the field will need better interoperability, not only across tools but across levels of inference. Functional studies should state explicitly whether they report capacity, realization, or impact; harmonize ontologies and metadata; preserve uncertainty in machine-readable forms; and make unresolved annotations visible rather than discarding them. The unresolved fraction of microbiome function is not merely missing data, but a biologically meaningful space of unknowns that should remain part of interpretation.

The long-term goal of microbiome functional research should therefore be to build predictive, experimentally grounded, and ecologically informed models that connect encoded capacity to realized activity and downstream impact.

## Supplementary Information

Below is the link to the electronic supplementary material.

Supplementary Material 1

## Data Availability

No datasets were generated or analysed during the current study.

## References

[1] McGuinness AJ, et al. From hype to hope: considerations in conducting robust microbiome science. Brain Behav Immun. 2024;115:120–30. 10.1016/j.bbi.2023.09.022. 37806533

[2] Djemiel C, et al. Inferring microbiota functions from taxonomic genes: a review. GigaScience. 2022;11. 10.1093/gigascience/giab090. PMC8756179 35022702

[3] Williams CE, Hammer TJ, Williams CL. Diversity alone does not reliably indicate the healthiness of an animal microbiome. ISME J. 2024;18. 10.1093/ismejo/wrae133. PMC11334719 39018234

[4] Thinnes CC, et al. MicroMap: a network visualisation resource for human microbiome metabolism. NPJ Biofilms Microbiomes. 2025;11. 10.1038/s41522-025-00853-0. PMC12663093 41315289

[5] Moyne O, et al. Predicting competition and substrate preferences for targeted microbiome alteration. Cell. 2026. 10.1016/j.cell.2026.03.036. 41999742 PMC13105274

[6] Muñoz-Mérida A, Muñoz-Pajares AJ. Beyond genomes: functional signatures reveal evolutionary patterns across the tree of life. Mol Biol Evol. 2026;43. 10.1093/molbev/msag121. PMC13234848 42126263

[7] Cao M, Cowen LJ (2017) When should we not transfer functional annotation between sequence paralogs? Pac Symp Biocomput 22:15-26. 10.1142/9789813207813_0003 27896958

[8] Levé M, et al. Metabolomics and metagenomics in mice reveal the role of the gut microbiota in tryptophan metabolism. iScience. 2025;28:113751. 10.1016/j.isci.2025.113751. 41234773 PMC12606010

[9] Sulaiman JE, et al. Proteomic study of *Akkermansia muciniphila* and *Bifidobacterium* species co-culture under different carbon sources. Vol. 2025;16–2025. 10.3389/fmicb.2025.1666747. PMC12631272 41277960

[10] Chang Y-C, et al. COMBREX-DB: an experiment centered database of protein function: knowledge, predictions and knowledge gaps. Nucleic Acids Res. 2016;44:D330–5. 10.1093/nar/gkv1324. 26635392 PMC4702925

[11] Leale AM, Reyes Marquez F, Zwaan B, Smid EJ, Schoustra S. Shifts and rebound in microbial community function following repeated introduction of a novel species. Oikos. 2025:e10880. 10.1111/oik.10880.

[12] da Silva AC, Lapkin J, Yin Q, Muller E, Almeida A. Meta-analysis of the uncultured gut microbiome across 11,115 global metagenomes reveals a candidate signature of health. Cell Host Microbe. 2026;34:379–e392375. 10.1016/j.chom.2026.01.013. 41666920

[13] Thornburg ZR, et al. Bringing the genetically minimal cell to life on a computer in 4D. Cell. 2026;189:2582–e25972527. 10.1016/j.cell.2026.02.009. 41806832

[14] Louca S, et al. Function and functional redundancy in microbial systems. Nat Ecol Evol. 2018;2:936–43. 10.1038/s41559-018-0519-1. 29662222

[15] Fatemi S, et al. Microbial composition and function are nested and shaped by food web topologies. ISME Commun. 2025;5. 10.1093/ismeco/ycaf175. PMC12558044 41158324

[16] Chung HA, Fralish Z, Tu T, Reker D. Profiling biological effects of microbiome metabolites via machine learning. iScience. 2026;29:115282. 10.1016/j.isci.2026.115282. 41959668 PMC13059116

[17] Seemann T. Prokka: rapid prokaryotic genome annotation. Bioinformatics. 2014;30:2068–9. 10.1093/bioinformatics/btu153. 24642063

[18] Overbeek R, et al. The SEED and the Rapid Annotation of microbial genomes using Subsystems Technology (RAST). Nucleic Acids Res. 2014;42:D206–214. 10.1093/nar/gkt1226. 24293654 PMC3965101

[19] Tatusova T, et al. NCBI prokaryotic genome annotation pipeline. Nucleic Acids Res. 2016;44:6614–24. 10.1093/nar/gkw569. 27342282 PMC5001611

[20] Lu S, et al. CDD/SPARCLE: the conserved domain database in 2020. Nucleic Acids Res. 2020;48:D265–8. 10.1093/nar/gkz991. 31777944 PMC6943070

[21] Zhou S, Liu B, Zheng D, Chen L, Yang J. VFDB 2025: an integrated resource for exploring anti-virulence compounds. Nucleic Acids Res. 2025;53:D871–7. 10.1093/nar/gkae968. 39470738 PMC11701737

[22] Ontiveros-Palacios N, et al. Rfam 15: RNA families database in 2025. Nucleic Acids Res. 2025;53:D258–67. 10.1093/nar/gkae1023. 39526405 PMC11701678

[23] Huson DH, et al. MEGAN Community Edition - Interactive Exploration and Analysis of Large-Scale Microbiome Sequencing Data. PLoS Comput Biol. 2016;12:e1004957. 10.1371/journal.pcbi.1004957. 27327495 PMC4915700

[24] Quast C, et al. The SILVA ribosomal RNA gene database project: improved data processing and web-based tools. Nucleic Acids Res. 2013;41:D590–596. 10.1093/nar/gks1219. 23193283 PMC3531112

[25] Muth T, et al. MPA Portable: A Stand-Alone Software Package for Analyzing Metaproteome Samples on the Go. Anal Chem. 2018;90:685–9. 10.1021/acs.analchem.7b03544. 29215871 PMC5757220

[26] Mitchell AL, et al. InterPro in 2019: improving coverage, classification and access to protein sequence annotations. Nucleic Acids Res. 2019;47:D351–60. 10.1093/nar/gky1100. 30398656 PMC6323941

[27] Singh G. Unipept 4.0: Functional Analysis of Metaproteome Data. J Proteome Res. 2019;18:606–15. 10.1021/acs.jproteome.8b00716. 30465426

[28] Shaffer M, et al. DRAM for distilling microbial metabolism to automate the curation of microbiome function. Nucleic Acids Res. 2020;48:8883–900. 10.1093/nar/gkaa621. 32766782 PMC7498326

[29] Lombard V, Ramulu G, Drula H, Coutinho E, P. M., Henrissat B. The carbohydrate-active enzymes database (CAZy) in 2013. Nucleic Acids Res. 2014;42:D490–495. 10.1093/nar/gkt1178. 24270786 PMC3965031

[30] Kanehisa M, Sato Y. KEGG Mapper for inferring cellular functions from protein sequences. Protein science: publication Protein Soc. 2020;29:28–35. 10.1002/pro.3711. PMC6933857 31423653

[31] Albrecht B, Bağcı C, Huson DH. MAIRA- real-time taxonomic and functional analysis of long reads on a laptop. BMC Bioinf suppl. 2020;13 21:1–12. 10.1186/s12859-020-03684-2. PMC7495841 32938391

[32] Alcock BP, et al. CARD 2023: expanded curation, support for machine learning, and resistome prediction at the Comprehensive Antibiotic Resistance Database. Nucleic Acids Res. 2023;51:D690–9. 10.1093/nar/gkac920. 36263822 PMC9825576

[33] Eng A, Verster AJ, Borenstein E. MetaLAFFA: a flexible, end-to-end, distributed computing-compatible metagenomic functional annotation pipeline. BMC Bioinformatics. 2020;21:471. 10.1186/s12859-020-03815-9. 33087062 PMC7579964

[34] David L, Vicedomini R, Richard H, Carbone A. Targeted domain assembly for fast functional profiling of metagenomic datasets with S3A. Bioinf (Oxford England). 2020;36:3975–81. 10.1093/bioinformatics/btaa272. PMC7332565 32330240

[35] Beghini F, et al. Integrating taxonomic, functional, and strain-level profiling of diverse microbial communities with bioBakery 3. eLife. 2021;10. 10.7554/eLife.65088. PMC8096432 33944776

[36] Schwengers O, et al. Bakta: rapid and standardized annotation of bacterial genomes via alignment-free sequence identification. Microb Genom. 2021;7. 10.1099/mgen.0.000685. PMC8743544 34739369

[37] Norouzi-Beirami MH, Marashi S-A, Banaei-Moghaddam AM, Kavousi K. CAMAMED: a pipeline for composition-aware mapping-based analysis of metagenomic data. NAR Genom Bioinform. 2021;3. 10.1093/nargab/lqaa107. PMC7787360 33575649

[38] Cantalapiedra CP, Hernández-Plaza A, Letunic I, Bork P, Huerta-Cepas J. eggNOG-mapper v2: Functional Annotation, Orthology Assignments, and Domain Prediction at the Metagenomic Scale. Mol Biol Evol. 2021;38:5825–9. 10.1093/molbev/msab293. 34597405 PMC8662613

[39] Queirós P, Delogu F, Hickl O, May P, Wilmes P. Mantis: flexible and consensus-driven genome annotation. Gigascience. 2021;10. 10.1093/gigascience/giab042. PMC8170692 34076241

[40] Milani C, et al. METAnnotatorX2: a Comprehensive Tool for Deep and Shallow Metagenomic Data Set Analyses. mSystems. 2021;6:101128msystems0058321. 10.1128/mSystems.00583-21. 34184911 PMC8269244

[41] Ruiz-Perez CA, Conrad RE, Konstantinidis KT. MicrobeAnnotator: a user-friendly, comprehensive functional annotation pipeline for microbial genomes. BMC Bioinformatics. 2021;22:11. 10.1186/s12859-020-03940-5. 33407081 PMC7789693

[42] Bardou P, et al. MINTIA: a metagenomic INserT integrated assembly and annotation tool. PeerJ. 2021;9:e11885. 10.7717/peerj.11885. 34692239 PMC8483015

[43] Vicedomini R, Blachon C, Oteri F, Carbone A. MyCLADE: a multi-source domain annotation server for sequence functional exploration. Nucleic Acids Res. 2021;49:W452–8. 10.1093/nar/gkab395. 34023906 PMC8262732

[44] Tyagi P, Bhide M. Development of a bioinformatics platform for analysis of quantitative transcriptomics and proteomics data: the OMnalysis. PeerJ. 2021;9:e12415. 10.7717/peerj.12415. 34820180 PMC8588854

[45] Mora-Márquez F, Chano V, Vázquez-Poletti JL. López de Heredia, U. TOA: A software package for automated functional annotation in non-model plant species. Mol Ecol Resour. 2021;21:621–36. 10.1111/1755-0998.13285. 33070442

[46] Maia GA, et al. AnnotaPipeline: an integrated tool to annotate eukaryotic proteins using multi-omics data. Front Genet. 2022;13–2022. 10.3389/fgene.2022.1020100. PMC9723129 36482896

[47] Dietrich A, et al. Namco: a microbiome explorer. Microb Genom. 2022;8. 10.1099/mgen.0.000852. PMC9484756 35917163

[48] de Jong A, Kuipers OP, Kok J. FUNAGE-Pro: comprehensive web server for gene set enrichment analysis of prokaryotes. Nucleic Acids Res. 2022;50:W330–6. 10.1093/nar/gkac441. 35641095 PMC9252808

[49] Sequeira JC, Rocha M, Alves MM, Salvador AF, UPIMAPI. reCOGnizer and KEGGCharter: Bioinformatics tools for functional annotation and visualization of (meta)-omics datasets. Comput Struct Biotechnol J. 2022;20:1798–810. 10.1016/j.csbj.2022.03.042. 35495109 PMC9034014

[50] Tatusov RL, et al. The COG database: an updated version includes eukaryotes. BMC Bioinformatics. 2003;4. 10.1186/1471-2105-4-41. PMC222959 12969510

[51] Wu J, et al. HiFun: homology independent protein function prediction by a novel protein-language self-attention model. Brief Bioinform. 2023;24. 10.1093/bib/bbad311. 37649370

[52] Lopez-Ibañez J, Pazos F, Chagoyen M. MBROLE3: improved functional enrichment of chemical compounds for metabolomics data analysis. Nucleic Acids Res. 2023;51:W305–9. 10.1093/nar/gkad405. 37178003 PMC10320153

[53] Porcheddu M, Abbondio M, De Diego L, Uzzau S, Tanca A. Meta4P: A User-Friendly Tool to Parse Label-Free Quantitative Metaproteomic Data and Taxonomic/Functional Annotations. J Proteome Res. 2023;22:2109–13. 10.1021/acs.jproteome.2c00803. 37116187 PMC10243107

[54] Cheng K, et al. MetaLab-MAG: A Metaproteomic Data Analysis Platform for Genome-Level Characterization of Microbiomes from the Metagenome-Assembled Genomes Database. J Proteome Res. 2023;22:387–98. 10.1021/acs.jproteome.2c00554. 36508259 PMC9903328

[55] Richardson L, et al. MGnify: the microbiome sequence data analysis resource in 2023. Nucleic Acids Res. 2023;51:D753–9. 10.1093/nar/gkac1080. 36477304 PMC9825492

[56] Vishwakarma A, Padmashali N, Thiyagarajan S, AnnoDUF:. A Web-Based Tool for Annotating Functions of Proteins Having Domains of Unknown Function. J Proteome Res. 2024;23:4296–302. 10.1021/acs.jproteome.4c00251. 39215721

[57] Pang Z, et al. MetaboAnalyst 6.0: towards a unified platform for metabolomics data processing, analysis and interpretation. Nucleic Acids Res. 2024;52:W398–406. 10.1093/nar/gkae253. 38587201 PMC11223798

[58] Sequeira JC, et al. MOSCA 2.0: A bioinformatics framework for metagenomics, metatranscriptomics and metaproteomics data analysis and visualization. Mol Ecol Resour. 2024;24:e13996. 10.1111/1755-0998.13996. 39099161

[59] Leblanc S, et al. OpenProt 2.0 builds a path to the functional characterization of alternative proteins. Nucleic Acids Res. 2024;52:D522–8. 10.1093/nar/gkad1050. 37956315 PMC10767855

[60] Gurbich TA, Beracochea M, De Silva NH, Finn RD. mettannotator: a comprehensive and scalable Nextflow annotation pipeline for prokaryotic assemblies. Bioinf (Oxford England). 2025;41:btaf037. 10.1093/bioinformatics/btaf037. PMC11842068 39854281

[61] Li Z, et al. ProtPipe: A Multifunctional Data Analysis Pipeline for Proteomics and Peptidomics. Genomics Proteom Bioinf. 2025;22:qzae083. 10.1093/gpbjnl/qzae083. PMC11842048 39576693

[62] Xia Y, et al. MetaflowX: a scalable and resource-efficient workflow for multi-strategy metagenomic analysis. Nucleic Acids Res. 2025;53. 10.1093/nar/gkaf954. PMC12489473 41036626

[63] Ghozlane A, et al. Accurate profiling of microbial communities for shotgun metagenomic sequencing with Meteor2. Microbiome. 2025;13:227. 10.1186/s40168-025-02249-w. 41199348 PMC12590682

[64] Bring Horvath ER, Winter JM. SeqForge: a scalable platform for alignment-based searches, motif detection, and sequence curation across meta/genomic datasets. BMC Bioinformatics. 2025;26:280. 10.1186/s12859-025-06297-9. 41254499 PMC12625553

[65] Chen W, et al. TraceMetrix: a traceable metabolomics interactive analysis platform. J Cheminform. 2025;17. 10.1186/s13321-025-01095-0. PMC12481999 41024283

[66] Aminu S, Ascandari A, Benhida R, Daoud R. GRUMB: a genome-resolved metagenomic framework for monitoring urban microbiomes and diagnosing pathogen risk. Bioinf (Oxford England). 2025;41:btaf548. 10.1093/bioinformatics/btaf548. PMC12548037 41002268

[67] Bruscadin JJ, et al. HolomiRA: a reproducible pipeline for miRNA binding site prediction in microbial genomes. BMC Bioinformatics. 2025;26:236. 10.1186/s12859-025-06241-x. 41034705 PMC12487068

[68] Rodrigues GVP, Ferreira LYM, Aguiar ERGR. ViralQuest: a user-friendly interactive pipeline for viral-sequences analysis and curation. BMC Bioinformatics. 2026;27:64. 10.1186/s12859-026-06391-6. 41667950 PMC12990509

[69] Douglas GM, et al. PICRUSt2 for prediction of metagenome functions. Nat Biotechnol. 2020;38:685–8. 10.1038/s41587-020-0548-6. 32483366 PMC7365738

[70] Altschul SF, Gish W, Miller W, Myers EW, Lipman DJ. Basic local alignment search tool. J Mol Biol. 1990;215:403–10. 10.1016/s0022-2836(05)80360-2. 2231712

[71] Buchfink B, Xie C, Huson DH. Fast and sensitive protein alignment using DIAMOND. Nat Methods. 2015;12:59–60. 10.1038/nmeth.3176. 25402007

[72] Eddy SR. Profile hidden Markov models. Bioinformatics. 1998;14:755–63. 10.1093/bioinformatics/14.9.755. 9918945

[73] Steinegger M, Söding J. MMseqs2 enables sensitive protein sequence searching for the analysis of massive data sets. Nat Biotechnol. 2017;35:1026–8. 10.1038/nbt.3988. 29035372

[74] Boutet E, Lieberherr D, Tognolli M, Schneider M, Bairoch A. UniProtKB/Swiss-Prot. Methods Mol Biol. 2007;406:89–112. 10.1007/978-1-59745-535-0_4. 18287689

[75] Haft DH, et al. RefSeq: an update on prokaryotic genome annotation and curation. Nucleic Acids Res. 2018;46:D851–60. 10.1093/nar/gkx1068. 29112715 PMC5753331

[76] Kanehisa M, Goto S. KEGG: kyoto encyclopedia of genes and genomes. Nucleic Acids Res. 2000;28:27–30. 10.1093/nar/28.1.27. 10592173 PMC102409

[77] Karp PD, et al. The BioCyc collection of microbial genomes and metabolic pathways. Brief Bioinform. 2019;20:1085–93. 10.1093/bib/bbx085. 29447345 PMC6781571

[78] Gene Ontology Consortium. going forward. Nucleic Acids Res. 2015;43:D1049–1056. 10.1093/nar/gku1179. 25428369 PMC4383973

[79] Peracchi A. The Limits of Enzyme Specificity and the Evolution of Metabolism. Trends Biochem Sci. 2018;43:984–96. 10.1016/j.tibs.2018.09.015. 30472990

[80] Röttig M, Rausch C, Kohlbacher O. Combining structure and sequence information allows automated prediction of substrate specificities within enzyme families. PLoS Comput Biol. 2010;6:e1000636. 10.1371/journal.pcbi.1000636. 20072606 PMC2796266

[81] Mistry J et al. Pfam: The protein families database in 2021. J Nucleic Acids Res. 2021;49:D412-D419. 10.1093/nar/gkaa913. PMC7779014 33125078

[82] Letunic I, Bork P. SMART v10: three decades of the protein domain annotation resource. J Nucleic Acids Res. 2026;54:D499-D503. 10.1093/nar/gkaf1023 PMC12807790 41062452

[83] Haft DH, Selengut JD, White O. The TIGRFAMs database of protein families. Nucleic Acids Res. 2003;31:371–3. 10.1093/nar/gkg128. 12520025 PMC165575

[84] Galperin MY, et al. COG database update: focus on microbial diversity, model organisms, and widespread pathogens. Nucleic Acids Res. 2021;49:D274–81. 10.1093/nar/gkaa1018. 33167031 PMC7778934

[85] McDowall J, Hunter S. InterPro protein classification. Methods Mol Biol. 2011;694:37–47. 10.1007/978-1-60761-977-2_3. 21082426

[86] Gabriel L, et al. BRAKER3: Fully automated genome annotation using RNA-seq and protein evidence with GeneMark-ETP, AUGUSTUS, and TSEBRA. Genome Res. 2024;34:769–77. 10.1101/gr.278090.123. 38866550 PMC11216308

[87] Brůna T, Lomsadze A, Borodovsky M. GeneMark-ETP significantly improves the accuracy of automatic annotation of large eukaryotic genomes. Genome Res. 2024;34:757–68. 10.1101/gr.278373.123. 38866548 PMC11216313

[88] Nomburg J, et al. Birth of protein folds and functions in the virome. Nature. 2024;633:710–7. 10.1038/s41586-024-07809-y. 39187718 PMC11410667

[89] Shaffer M, et al. DRAM for distilling microbial metabolism to automate the curation of microbiome function. Nucleic Acids Res. 2020;48:8883–900. 10.1093/nar/gkaa621. 32766782 PMC7498326

[90] Jumper J, et al. Highly accurate protein structure prediction with AlphaFold. Nature. 2021;596:583–9. 10.1038/s41586-021-03819-2. 34265844 PMC8371605

[91] Yu T, et al. Enzyme function prediction using contrastive learning. Science. 2023;379:1358–63. 10.1126/science.adf2465. 36996195

[92] Armengaud J. Metaproteomics to understand how microbiota function: The crystal ball predicts a promising future. Environ Microbiol. 2023;25:115–25. 10.1111/1462-2920.16238. 36209500 PMC10091800

[93] Montero-Blay A, Piñero-Lambea C, Miravet-Verde S, Lluch-Senar M, Serrano L. Inferring Active Metabolic Pathways from Proteomics and Essentiality Data. Cell Rep. 2020;31:107722. 10.1016/j.celrep.2020.107722. 32492430 PMC7273199

[94] Ammer-Herrmenau C, et al. Gut microbiota predicts severity and reveals novel metabolic signatures in acute pancreatitis. Gut. 2024;73:485. 10.1136/gutjnl-2023-330987. 38129103 PMC10894816

[95] Jia Y, et al. Integrating metagenomics with metabolomics for gut microbiota and metabolites profiling in acute pancreatitis. Sci Rep. 2024;14:21491. 10.1038/s41598-024-72057-z. 39277616 PMC11401878

[96] Vanni C, et al. Unifying the known and unknown microbial coding sequence space. eLife. 2022;11. 10.7554/eLife.67667. PMC9132574 35356891

[97] Almeida A, et al. A unified catalog of 204,938 reference genomes from the human gut microbiome. Nat Biotechnol. 2021;39:105–14. 10.1038/s41587-020-0603-3. 32690973 PMC7801254

[98] Nolan LM, Webber MA, Filloux A. Throwing a spotlight on genomic dark matter: The power and potential of transposon-insertion sequencing. J Biol Chem. 2025;301:110231. 10.1016/j.jbc.2025.110231. 40378959 PMC12173739

[99] Méheust R, Burstein D, Castelle CJ, Banfield JF. The distinction of CPR bacteria from other bacteria based on protein family content. Nat Commun. 2019;10:4173. 10.1038/s41467-019-12171-z. 31519891 PMC6744442

[100] Salazar G, et al. Gene Expression Changes and Community Turnover Differentially Shape the Global Ocean Metatranscriptome. Cell. 2019;179:1068–e10831021. 10.1016/j.cell.2019.10.014. 31730850 PMC6912165

[101] Ma B, et al. A genomic catalogue of soil microbiomes boosts mining of biodiversity and genetic resources. Nat Commun. 2023;14:7318. 10.1038/s41467-023-43000-z. 37951952 PMC10640626

[102] Van Den Bossche T, et al. The microbiologist’s guide to metaproteomics. iMeta. 2025;4:e70031. 10.1002/imt2.70031. 40469504 PMC12130581

[103] Dumas T, et al. The astounding exhaustiveness and speed of the Astral mass analyzer for highly complex samples is a quantum leap in the functional analysis of microbiomes. Microbiome. 2024;12:46. 10.1186/s40168-024-01766-4. 38454512 PMC10918999

[104] Zhou X, et al. Longitudinal profiling of the microbiome at four body sites reveals core stability and individualized dynamics during health and disease. Cell Host Microbe. 2024;32:506–e526509. 10.1016/j.chom.2024.02.012. 38479397 PMC11022754

[105] Liu X, Salles JF. Drivers and consequences of microbial community coalescence. ISME J. 2024;18. 10.1093/ismejo/wrae179. PMC11447283 39288091

[106] Tropini C, Earle KA, Huang KC, Sonnenburg JL. The Gut Microbiome: Connecting Spatial Organization to Function. Cell Host Microbe. 2017;21:433–42. 10.1016/j.chom.2017.03.010. 28407481 PMC5576359

[107] Ramond P, Galand PE, Logares R. Microbial functional diversity and redundancy: moving forward. FEMS Microbiol Rev. 2025;49. 10.1093/femsre/fuae031. PMC11756291 39689915

[108] Gopalakrishnappa C, Gowda K, Prabhakara KH, Kuehn S. An ensemble approach to the structure-function problem in microbial communities. iScience. 2022;25:103761. 10.1016/j.isci.2022.103761. 35141504 PMC8810406

[109] Sun B, et al. Metaproteomics Reveals Community Coalescence Outcomes in Co-Cultured Human Gut Microbiota. Proteomics. 2025;25:6–18. 10.1002/pmic.70009. 40713811

[110] Hong J, Xue W, Wang T. Universal gene-level bimodality in natural microbial communities. Cell Rep. 2026;45. 10.1016/j.celrep.2026.117013. 41712385

[111] Moraïs S, et al. Community context reshapes microbial proteomes and reduces functional overlap. Nat Microbiol. 2026. 10.1038/s41564-026-02310-w. 42032280 PMC13171630

[112] Zhai F, Zhang C, Yu X, Hu B, Shan B. Microbial ecological networks reveal community reorganization along a sediment biotoxicity gradient. Ecotoxicol Environ Saf. 2026;317:120172. 10.1016/j.ecoenv.2026.120172. 42066444

