# Supplementary material for: From functional annotation to functional meaning in microbiome research
Source: Environ Microbiome. 2026 Sep 2;21:108. doi: 10.1186/s40793-026-00956-x (PMC13587530; doi:10.1186/s40793-026-00956-x)
Supplement: Supplementary file 1 [file 40793_2026_956_MOESM1_ESM.docx]

This systematic review aimed to identify functional annotation tools through a targeted literature search of the PubMed database (https://pubmed.ncbi.nlm.nih.gov/), covering publications from 2020 to 2026. Three Boolean search strategies were developed to capture (i) terms related to functional annotation (e.g., "functional annotat*", "genome annotat*"), (ii) terms associated with bioinformatics tools and platforms (e.g., "softwar*", "web-server*"), and (iii) terms representing multi-omics approaches (e.g., "microbiome", "metagenomics", "metabolomics"). The three search strings were combined using the Boolean AND operator, yielding 716 records, with filters applied to exclude reviews, preprints, non-English publications, and articles lacking full-text availability.

Titles and abstracts of the retrieved records were screened against predefined eligibility criteria, excluding: (1) studies not presenting a tool or software; (2) tools not designed for functional annotation (e.g., purely data-handling or processing utilities); (3) duplicate tools; (4) inaccessible or non-functional tools or websites; (5) tools developed exclusively for narrow, case-specific applications; and (6) studies merely applying previously published annotation tools rather than introducing new ones.

This screening process identified 35 functional annotation tools meeting the eligibility criteria. To enhance comprehensiveness, citation searching was used to identify an additional 11 widely referenced tools; RAST, Prokka, Mantis, NCBI-PGAP, InterPro, MetaProteomeAnalyzer (MPA), CLEAN, MicrobeAnnotator, HUMAnN 3.0, MEGAN6 and PICRUSt2 that were frequently cited across the selected studies. In total, 46 functional annotation tools were curated for the analysis.

**Figure S1.** PRISMA flowchart depicting the systematic literature review process for identifying functional annotation tools in microbiome research.
